# “Transgenders are not dinosaurs!” Stigma faced by transgender women in their daily lives in India: implications for research and policy

**DOI:** 10.12688/wellcomeopenres.22658.1

**Published:** 2024-09-03

**Authors:** Sandhya Kanaka Yatirajula, Ankita Mukherjee, Santosh Giri, Pallab K Maulik

**Affiliations:** 1 The George Institute for Global Health India, New Delhi, Delhi, 110025, India; 2 Kolkata Rista, Kolkata, West Bengal, 700054, India; 3 University of New South Wales, Sydney, New South Wales, 2052, Australia; 4 Imperial College London, London, England, SW7 2AZ, UK; 5 Prasanna School of Public Health, Manipal Academy of Higher Education, Manipal, Karnataka, 576104, India

**Keywords:** Transwomen, mental wellbeing, stigma, India

## Abstract

**Background:**

Transgender women face stigma that adversely impacts their mental wellbeing. The stigma can be self-directed (internal), discrimination, violence and hatred directed towards them by others, mostly cis-gender persons (interpersonal stigma) and discrimination faced at the level of institutional arrangements (structural stigma).

**Methods:**

This was an exploratory study that used qualitative methods of data collection (focused group discussions and in-depth interviews) to gather data from consenting adult trans women who lived in the city of Kolkata situated in the eastern state of West Bengal in India.

**Results:**

The findings showed that trans women faced the trauma of non-acceptance and even rejection by their families when they began to express their chosen gender. Their gender non-conforming behaviour made them the butt of ridicule and harassment in school, resulting in many of them not finishing school. This made finding employment difficult. The trans women study participants also faced harassment at the hands of the police and from hospital staff, making them reluctant to approach the police for help and seek treatment from health providers for their physical as well as mental health concerns.

**Conclusions:**

It is important for researchers to be cognizant of the challenges faced by trans women/transgender people while designing and conducting research. It is also important for policymakers to make gender affirming policies to mitigate and eliminate the stigma that transgender people are subject to thereby promoting their wellbeing.

## Plain Language Summary

Most research on transgender women focused on HIV related issues; there is a lack of research that has focused on mental health of trans women globally, very few studies in low- and middle-income countries (LMICs) and even less in India. The existing research has shown that trans women experience depression, anxiety, and other psychiatric conditions, much more that cis-gender persons. Research has also shown that incidences of suicide and suicide ideation is much higher among this population when compared to cis-gender persons.

This perhaps is the only study in India that focuses on the stigma experienced by trans women, its implications for designing future research interventions and policies. It tries to provide insights into the challenges and struggles faced by them in their daily lives which has a bearing on identifying issues that are of greater relevance to the transgender community, which in turn should guide future research and policy interventions.

There are 4.8 million transgender persons in India, many of whom are unable to lead fulfilling lives due to the stigma and discrimination that they face. It is critical for more research to be done among them to identify issues that are of relevance to the community and that can feed into the development of evidence informed policies that are sensitive to their needs. Adequate investment in research on transgender people’s experiences, not just in India but the world over, will promote greater social inclusion, equity, and gender justice, thereby advancing the realisation of the Sustainable Development Goals.

## Introduction

Stigma has been defined as stereotypes or negative views attributed to a person or groups of people when their characteristics or behaviours are viewed as different from or inferior to societal norms ^1^. Transgender people experience a high level of social stigma, internalised stigma ^2, 3^ social isolation, and victimization ^4^. Stigma operates and is experienced at three levels – internal stigma, interpersonal stigma, and structural stigma. Internal stigma stems from the feelings that people have about themselves, or their perception of what others think of them. Interpersonal stigma refers to the treatment that individuals are subjected to by others because of their gender identity or gender expression. Structural stigma refers to the societal norms, environmental conditions, policies, institutional laws, and practices that may inhibit individuals and groups from reaching their potential ^2^. Transgender people experience all these three forms of stigma which adversely affects their wellbeing ^5– 7^. They face rejection from their families, and are subjected to ridicule, discrimination, and exploitation due to their gender variant behaviour and roles ^8^. The lack of acceptance and social exclusion adversely affects their self-esteem ^9^ resulting in depression, anxiety and psychological distress ^10– 12^.

One of the theories that has influenced research on health and well-being in sexual and gender minorities is the minority stress model propounded by Meyer who identified two major stressors (distal and proximal stressors) faced by sexual and gender minorities; these results in an excess stress burden on them, which adversely affects their mental wellbeing ^13^. Coping mechanisms, social support and resilience helps to mitigate the impact of these stressors ^14^. Resilience is closely related to mental wellbeing and helps individuals to thrive in the face of adversities ^15^. Research has shown that transgender people have low resilience when compared to cisgender individuals ^8^. Lower resilience puts individuals at a greater risk of mental health problems ^16^. Social support is a protective factor which helps develop resilience ^17^, as are, acceptance, education, employment, and social inclusion ^18^. Due to limited economic opportunities available to them, many trans women are forced to engage in sex work ^19^ and begging. Social and institutional stigma against them prevents them for accessing opportunities and affects their access to education, employment, and healthcare ^20, 21^.

## Methods

### Aims

The larger study, from where the data presented in this article has been taken, used qualitative methods to draw upon the lived experience and insights of transgender women with the following aims:

1.   To map the issues of involving trans women in mental health research

2.   To identify ethical principles to keep in mind while conducting research among transwomen

3.   To identify mental health research needs of trans women

### Methods

Qualitative methods were used to draw upon the lived experience and insights of trans women in order to map the issues of involving trans women in mental health research and to identify ethical requirements of conducting research among trans women. The ethical issues of conducting research among trans women has been discussed in a sister paper ^22^ and is not part of this article. This article focuses on the lived experience of trans women and its implications on planning research among transgender women.

This study was conducted with trans women only, as the collaborating partner, Kolkata Rista (KR), works almost exclusively with trans women. KR is a trans-led community-based organisation headquartered in Kolkata, a city in east India. The research team comprised of two cis-gender women, one of whom had earlier existing relationships with KR and therefore access to trans women became possible. Three focused group discussions (FGDs) and five in-depth interviews (IDIs) were conducted with 30 transgender women. The participants were purposely selected in consultation with Kolkata Rista. Data were collected between 7 ^th^ to 10 ^th^ November 2022. The FGDs and IDIs were conducted in the local language Bengali and the recordings were translated into English. SKY and AM reviewed the transcripts independently to identify codes, which were finalised following a discussion between them. The following codes were identified after which the data were thematically analysed using NVivo 12 ( Standard installation <NVivo_12_for_Windows_perpetual> (qsrinternational.com).

1. About *Hijras*
2. Family Background
  2.1 Family members
    2.1.2 Education and jobs of parents and partners
3. Identity
  3.1 Childhood experiences
  3.2 Realisation of trans identity - when & how
  3.3 Barriers for family acceptance
  3.4 Facilitators for family acceptance
  3.5 Family acceptance - positive attitudes and behaviours
  3.6 Family reaction to trans identity
  3.7 Perception of support from family, partners, others
  3.8 Getting surgery done
  3.9 Hormonal treatment
  3.10 Self affirmation
4. Stigma -
  4.1 Actual experience
  4.2 Societal attitude - public perception of trans women

Another data source that was drawn upon was the discussions that ensued during a public dissemination workshop held with trans women and other stakeholders to share the findings of the study. However, since formal consent was not taken before the workshop, no verbatims from the workshop are included in the analysis. Generic verbal consent was taken for recording the proceedings and using their photos in the dissemination report.

This study was approved by the Independent Ethics Committee of the George Institute for Global Health, New Delhi, India (Ref. no. 15/2022) on the 28 ^th^ July 2022.

### Participant selection

The study participants included adult trans women living in the city of Kolkata and included in the database of Kolkata Rista. Purposive selection was done based on willingness to participate and availability of participants.

## Results

### Experience of internal stigma/self-stigma

The trans women we spoke to mentioned that it takes a long time for trans women to understand and accept themselves and to come to terms with their trans-identity. The pressure to conform to societal standards was high. This leads to confusion, stress, insecurity, and gender dysphoria.

*“I used to think that why I am not like my elder brother! Like my elder brother used to play football, cricket and all that, or he was hanging out with his male friends, so why can't I be like them! Or when I saw my sister, I wondered why I can't be like my sister. My sister used to wear that frock and then the dresses that are meant for girls, she would wear kajal, mother would tie her hair. But mother is not doing the same with me! Because I want to be like that. So I used to feel very sad to see this. I would suffer. Sometimes I would sit down and cry to myself. Sometimes, God, I mean there was a photo of God in front of me, I used to see that photo of God and say ‘what have you done to me’ ”. –* PR

Some participants mentioned that they shunned company and tried to keep to themselves to avoid being shamed and judged, but that led to loneliness.

*“I would also need to talk or mingle with people. I mean how long can a person be alone!”* - HO

One of the ways in which participants were able to come to terms with their identity was through connecting with other trans women. Some participants spoke of support groups (community) that helped them to build a sense identity, confidence and empowerment, which has enabled many trans women to discover themselves and stand up for their rights. The participants mentioned that when they met others like themselves, they felt a sense of community and belongingness.

### Family acceptance

Acceptance of trans-identity among family members did not come easily. It usually is a struggle for both family members and the child who is beginning to identify as trans. Childhood experiences for many of the trans women respondents was challenging in the family setting and surfaced around puberty. On learning about the identify of their children/siblings, families reacted with disbelief, anger, or sadness.

*“When I told them [about my trans-identity] for the first time, everyone in my house went into depression”* – SA

Sometimes family members even resorted to violence.

*“I still remember that incident, as I was like this [wearing frocks and makeup] when I was a child, my father beat me with the wooden sticks. I didn't eat food for two days. It seemed like I should even commit suicide, so that was it”* – HO

Denial was common and some families resorted to seeking various avenues - astrologers, gurus, traditional healers - to find a ‘cure’ for their gender non-conforming child.

*“Many people try to do some black magic, parents take them, and many elders take them to an astrologer in the hope that their son would be fine”* - FGD 1433

Socialising with those considered as effeminate could also elicit negative reactions including being reprimanded and physically punished. There was fear that socialising with such effeminate boys would strengthen the perceived femineity among their own children. The absence of familial support was a major stressor for trans women that deeply affected their mental wellbeing. With time, there was some degree of acceptance, however the level of acceptance varied and might be from only a few of the family members. One of the respondents reported that while she can visit her family now and no questions are asked, yet she feels that her family is not totally comfortable with her trans-identity.

*Now, when I go home, the people around me don't even talk to me that much. I don't know why they don’t talk, I don't like it. Support has not been there since childhood. I would still say that there is no huge support even now. Even though I am doing so much for the family. Maybe I feel that there is some dilemma in the behaviour of my family.* – HO

The trans women we spoke to identified certain factors which facilitated family acceptance of their trans-identity. Often the money provided to the family helped in gaining acceptance. This made some of the participants feel sad.

*“It is also a matter of sorrow that the parents or brothers and sisters who could not accept me from the beginning, did not like me, but today they like me because of my money. What if I don't have income or can't pay after some time?”* - PR

Very few families have been supportive and were able to accept their child’s identity as trans.

*“They have not asked me to leave the house. I have not felt any discrimination from them. My parents said, ‘you are trans for your feelings, but you have to earn like a normal person. You are transgender, and we will prioritize your feelings, and accept your being’* ” - SA

Here the family was concerned that their son would get involved in sex work. Hence the emphasis on ‘earning like a normal person’. This participant currently stays with her family and is self-employed.

### Membership in a *Hijra gharana*

Some of the study participants were or had been members of *Hijra gharanas.* The *hijra* community in India are systematically organized as hierarchical communities within themselves, which are called *gharanas*. The word *hijra* is traditionally used to refer to trans women or intersex persons. It should be noted however that not all trans women identify themselves as or belong to the *hijra* community. The word *gharana means* household/community. Traditionally the *hijra* community visit a household during weddings or birth of a child to perform dances and give blessings and get money from the family in return. Each *gharana* is headed by a *Guru* (leader), who assumes the status of ‘mother’; the status is achieved when a senior trans woman receives a newly joined trans woman as her *chela* (follower/disciples). The *chela* has to serve the *Guru* and offer a certain portion of her daily earnings to her *Guru*. The *Guru* is the caretaker of her c *helas and* members of a gharana lived together under the patronage of their *Guru.* Those study participants who were members of a *Hijra gharana* in the past spoke of the benefits associated with being a member which was related to old age security - when the *Guru* becomes old, the *chelas* look after their *Guru*.

*“They (chelas) take care (of their Guru). Why? She (Guru) also worked for a time (in the past). At one time she also earned the bread. So those below her (chelas) take care (of the Guru) in their old age” -* PR

But these study participants also spoke of the existence of a hierarchy within the *gharana* based on differential power. The *Guru* is the most powerful and the newest *chela* is the least powerful. The *Guru* has the power to allocate *badhai* (payment given for traditional dancing and blessings showered by *Hijras* during weddings or the birth of a child, especially a son) or *challa* (begging) to their *chelas*. Being in a *Hijra* *gharana* one needs to follow the hierarchical norms. *Hijras* higher up in the hierarchy have certain privileges including being paid a share of the earnings. Both these were identified as sources of mental stress for those lower in the hierarchy. Giving up a share of their earnings meant that the *chelas* are left with meagre portion of their earnings after a hard day’s work in the sun and on the streets.

*“Now, those who are going to the badhai, they go around the whole neighbourhood to find out where [household] the baby was born. They get burnt in the hot sun and then bring the badhai. What happens when they return is that the Guru, who was sitting at home in the cool AC, takes half of the income that they earned after roaming around in the sun doing badhai or challa. This is a pressure that I worked all day, but where did my profit go?”* - PR

Another cause for stress was identified to be territorial disputes between *gharanas* which may turn violent. Some of the trans women participants mentioned that they had given up the membership of their *Hijra gharanas*. The reasons for leaving include the hierarchical power relations, lack of respect, existence of many rules, having to paying hefty fines ( *don khan*) for breaking the rules, and better employment opportunities that offered more freedom. Some of the rules in *gharanas* were perceived as being arbitrary. Fines had to be paid to senior members if the seniors felt that they had been disrespected in any way. Continuous fear of offending seniors and having to pay fines was identified as a major stressor by some of the trans women participants we spoke to.

*“Then they (senior trans women) will complain, they will complain a lot when there is some mistake in work... See there are a lot of rules, as if it is a separate world... They make rules among themselves, if something happens there, you have to pay don. Suppose you are my Guru, and I said something wrong to you then I have to give a don, or suppose your hair has grown, and you cut the hair… they may come up with a rule suddenly, and I will have to pay a don because only if my Guru gives permission, then I can cut my hair”* – HO

While some of the participants identified themselves as belonging to the *hijra* community, others were categorical in denying that they were *hijras.* This is because the term *hijra* has often been used derogatorily or pejoratively in common parlance. Some participants wanted to distance themselves from this. They emphasised that unlike common perception, every trans woman was not a *hijra*, as this specifically referred to a group that was part of a *gharana*, and followed the traditional occupation.

### Experience of structural and interpersonal stigma in educational institutions

The trans women respondents mentioned that attending school was a traumatic experience. Classmates would avoid interacting with them. They faced ridicule from their classmates, especially the boys who would refer to them as ‘girl design’. Some of them mentioned being harassed by teachers and school authorities on account of their gender expression.

*“One by one we [students perceived as being effeminate] were made to stand [by the teacher]. Then somewhere we felt insulted, we had no value by ourselves, there was no value for our studies [academic achievements], for what we were doing or that we were in a rank [top achievers], only our behaviour [effeminate] was being judged”* - PR

Some of the respondents mentioned that they were subjected to sexual abuse by students and even school authorities but were either too afraid to report it or were persuaded not to report the incident.

*“It also happened that he took me to the bathroom and abused me.... I would never feel safe going to school. I felt like someone was going to abuse me. This is one of the biggest issues”* – 1316 FGD

Others mentioned that they were able to cope with such situations because a few of the teachers were supportive of them, while some others mentioned finding solace in others trans-children like them at school.

*“Since I was a bit quiet …, I never used to run around like other boys and girls, so the teacher madams, they used to love me. So, I was given a place on a bench that I would sit on …, I never had to fight for a place to sit like others, I liked this thing as I was good at studies. So there was a place where I had to sit. Even in school, there have been many taunting-teasing incidents, but I have never been harassed by teachers. The teachers, I mean, they never said anything like that, girl design or anything like that”* – HO

However, such support was limited and many of the trans women mentioned that they were forced to discontinue their education on account of the treatment meted out to them in educational institutions. The respondents mentioned that the lack of education limited the opportunities available to them and severely affected their chances of securing well-paid jobs.

*“There is very little education within the trans community. I mean they are not educated. Because the pressure in college is the same as in school. The teachers are also the same. So, if they are not educated properly, they have to fight for their whole life”* – BA

The respondents suggested that two strategies would help to address the treatment meted out to them at school and would ensure that trans children did not feel forced to drop out of school in future. The first was to train teachers on how to deal with gender non-conforming children:

*“But I couldn't even tell the teachers [about being sexually abused in school], because they [teachers] would say that you are like a girl [effeminate]. So, I think that the training that the teachers have, should be about how to deal with a child” –* 1316 FGD

The second was to teach about gender non-conformity and what it meant in school. In the opinion of one of the respondents, such knowledge would also help gender non-conforming children to understand themselves better and would help cis-gender children to be more accepting of gender non-conforming children.

*“One more thing, in school, I think that the education that we first have,… means in the syllabus, means in the books, if any education is given to them [students] about this gender identity through books…If that knowledge is included in the syllabus books of the eighth, ninth class, like gender means this, sex means this, gender identity, what transgender is... these things. If all of these are given in one chapter in one syllabus, not much time, then the trans-child will have knowledge. And if they [cis-gender children] have that taught to them, they may not taunt them [gender non-conforming children]. It [discrimination] will decrease a little more, when compared to before, even now it has decreased a lot, I think it will decrease further*” - HO

### Experience of interpersonal stigma and discrimination in public spaces

The trans women we spoke to mentioned that they felt that society did not accept them, and they were subjected to discrimination in many places. Discrimination in public transportation was common - sometimes they were physically harassed, another respondent mentioned that often people snickered when they saw trans women or they turned their faces away, and many people were reluctant to sit next to them, fearing that the trans women would somehow cast a spell on them.

*“ I remember one of my friends faced an incident in a train. Someone in the train, told a kid [after spotting the friend] ‘look, look, aunty [Used with a negative connotation in this instance]. These aunties have powder in the bag, they will sprinkle it on you, and you will also become like aunties’ ”*

A few respondents mentioned that they were ill-treated on streets when they were going about their business or were begging.

*“When a parent is walking with their child, they try to keep their child away from us. This shouldn’t happen”* – 1433 FGD
*“Also, those of us who are begging, are treated very badly by some people on the street… Some people turn their faces away. These things have to change”* – 1316 FGD

The trans women told us that there was no acceptance or safety; they were subjected to sexual abuse and physical abuse sometimes in their own neighbourhoods. People are reluctant to have transgender people as neighbours and they are shunned from community celebrations.

“ *I went to Durga Pujo (religious festival) with my family. There I faced many abusive comments. My mom is blind. She had to listen to many bad things. They are worshipping a goddess [Durga], and in the same place, they are abusing a woman” –* AR

Some of the trans women reflected on the irony of the situation, as they are mostly ignored and illtreated, but at times they were sought after.

*“They don't accept us on the bus, on the platform, or the street, but whenever it comes to election, they* [political representatives trying to get votes from the transgender persons during elections] *accept us.” 1433 FGD*

### Experiences of structural stigma: the police and health providers

Structural stigma is pervasive and affects the mental wellbeing of transgender people. The stigma manifests itself in many ways such as lack of basic facilities for transgender people in public places (no separate toilets, no dedicated beds in hospitals). It also manifests itself in the way in which people working in public institutions responded to trans women and the perceptions that these providers have of trans women. The trans women mentioned that the police did not treat them with respect and even harassed them. The police would not be willing to register complaints brought to them by the trans women and believed that trans women themselves were the ones at fault.

*“…another part of society is the police. We have to suffer a lot from them. If there is any torture on us, and then, if we go to the police station, they don’t respect us. They think we are low-class people. They don't understand transgender. They understand ‘Hijre’ –* 1316 FGD
*“The police will not take any of our cases. They just file a GD [general diary]. If I say that one of us has been raped, they will say, ‘You too are raped!’ They start with this. The police ask about our rate [for sex work]. They don’t give us proper respect”* – 1316 FGD

Structural stigma also manifests itself in the terminology that is used even by the development sector to refer to transgender people. One of the respondents mentioned that in the HIV targeted intervention projects, one of the high-risk groups identified were female sex workers. But when it came to them (trans women), the term used was TG (transgenders), as if to imply that all transgender people were sex workers. The respondents felt that the term transgender sex workers should be used instead, as all trans women were not engaged in sex work. This insensitivity in terminology was a source of mental stress for the trans women.

*“Our social structure is wrong. In the TI [targeted intervention], it says FSW [Female Sex Worker]. And what is used to refer to us? TG [Transgender]! Why? Do all TGs do sex work? Write TG Sex Worker…These myths must be broken. They think that transgender means dirty. Why? This is wrong” –* 1316 FGD

The respondents mentioned that the medical fraternity also had biases against them and the experience of going to the hospital to seek treatment was fraught with humiliation. Staff at the facilities questioned them and asked them why they had become trans and there was always confusion with which queue they should be standing in. Some respondents mentioned that doctors avoided touching them and would prescribe medication without physically examining them.

*“We face problems even after going to the doctor. An incident happened to me. Once I had a fever. When I went to the hospital, the doctor looked at me from a distance and asked what happened. Then I said that I have a fever. So, he prescribed some medicines without touching [examining] me. This happens often”* -1316 FGD

One of the requirements for undergoing hormonal treatment/surgery in the state of West Bengal is to undergo counselling and get certified by a counsellor. The transwomen we spoke to mentioned that often the counsellors themselves did not know how to behave with them. Even private counsellors were not adequately trained or sensitive.

*“I visited the private one [counsellor who issues the transgender certificate]. He called my parents and asked them to do proper treatment [to cure the person]… Then I realised that he knows nothing properly”* – 1245 FGD

### Inclusion in policy formulation and representation in government committees

Trans women at the dissemination workshop voiced the opinion that the transgender community was not able to influence policy makers which was why things on the ground took time to get implemented. Therefore, they felt that it was important that transgender representatives be part of the process of policy formulation, so that their concerns are included when policies are designed. They also mentioned that transgender representation in committees such as the Child Welfare Committee (CWC) or Rogi Kalyan Samitis (RKS, hospital management committees) was critical since transgender people would be able to better understand and help gender non-conforming individuals when compared to cis-gender committee members.

### Creation of safe spaces

Trans women at the workshop also talked about the importance of creating safe spaces for the community to enable them to freely discuss problems and interact with each other without any fear. Villages and small towns especially lacked safe spaces and transgender persons from such places faced many challenges as displaying their trans-identity was not acceptable and could be even dangerous.

## Discussion and conclusion

This article draws upon qualitative data collected to highlight the issues faced by trans women in their daily lives related to childhood experiences, realisation of trans-identity, family reactions to expressions of trans-identity, experience of abuse/violence and so on. It also identifies both interpersonal and structural stigma experienced by trans women at home, in schools, police stations, health facilities and other public places. 

This study, to the best of our knowledge, is perhaps the only study in India that focuses on the research implications of the challenges faced by trans women in their daily lives. It tries to provide insights into the struggles faced by trans women which have a bearing on identifying the things that are more relevant to the transgender community and therefore on what to research. Transgender people face stigma which operates at various levels that impacts their health ^2^. The findings of this study indicate that trans women face stigma at the internal level (self-stigma), interpersonal level and structural level. The findings resonate with the social-ecological model of stigma which recognises that stigma experience by transgender people operates at three levels - individual, interpersonal and structural – and adversely impacts their health and wellbeing by restricting access to opportunities and resources, thereby putting them at a disadvantage and perpetuating the stigma and discrimination that they encounter ^23^.

### Experience of Self stigma

Self-stigma has been recognised as resulting from the feelings that individuals have about themselves or their perception of how people view them. Self-stigma influences the ways in which individuals respond to others and influences the ways in which they interact with others including the anticipation and avoidance of discrimination ^24^. Previous research has found that self-stigma results in depression, anxiety, suicide ideation and attempts and substance abuse ^5^.

### Experience of interpersonal stigma within family settings

For good mental health, family support and acceptance are vital ^25^. Lack of family support has been found to, at times, take on extreme forms, including physical assault, misgendering, rejection and turning the transgender child out of the house ^26^ or trying to find a ‘cure’ for such children. Parents of gender non-conforming children are often subjected to tremendous pressure from society and relatives. Providing counselling to parents to deal with these pressures and to accept their gender non-conforming children is an important strategy for policy makers to consider.

### Experience of Interpersonal stigma outside family settings

Transgender people encounter stigma from settings that are external to the family; for instance, they face victimization in educational institutions ^27, 28^ and from the general public ^29^. While at school gender non-conforming children are confused; on the one hand they compare themselves with cis-gender children and feel that because they do not think/feel like cis-gender children, there is something wrong with them. On the other hand they are often subjected to harassment and bullying that is perpetrated by the other children or sometime even by the teachers. Many a times this harassment reaches such limits that the child decides to dropout of school. As a result, most individuals who are transgender are not able to complete their formal education which affected their employability ^30^. Another step that can promote retention in schools could be making changes in the school curriculum that acknowledges and normalises gender non-conformity and make cis-gender children aware of the possible stigma/challenges that their transgender peers might be exposed to. This will go a long way to create citizens who are more informed. Researchers who are planning school level interventions should keep the experiences and unique needs of cis- gender students in mind.

To reduce and prevent dropouts from happening, it is critical to improve the environment in school. Schools can provide psychological support to such children by employing the services of counsellors or other mental health professionals. Alongside this it is critical to sensitise cis-gender children to adopt behaviours that are supportive of gender non-conformity and to view it as normal rather than a transgression. By introducing such measures, schools can become safe spaces for gender non-conforming children to express their gender non-conformity. Schools also need to overcome their hesitancy in discussing sexuality, gender and gender non-conformity issues ^31^.

### Experiencing stigma from health personnel and law enforcement personnel

Healthcare for transgender populations is limited due to stigma among health professionals ^32^ and within health facilities ^33^. Research has also shown that many transgender people experience ill-treatment in hospitals ^34^ and this is the reason why they are reluctant to seek treatment for care that might be necessary, resulting in worsening of their health ^35^. It has been found that in many contexts, medical training does not include transgender health ^36^ and, as a result, health personnel do not have the skills to provide services to transgender people, although the importance of their role in mitigating the negative impact of stigma and prejudice, assisting transpersons with becoming comfortable with alternate gender expression, and facilitating the process of transitioning and coming out, has been recognised ^37^. 

Transgender people are perceived to be different and are often denigrated and subjected to harassment, bullying, and discrimination by the police ^38^. The police are a critical institution for transgender populations, but research has shown that the relationship between the police and transgender individuals is influenced by mistrust, stigma, and the anticipation of abuse ^39^ and that many transgender people across the world have experienced such biased treatment at the hands of law enforcement ^40^. For instance, our respondents mentioned that there was a clear difference in the way transgender people were treated vis-à-vis cis-gender people by police personnel.

The authors are of the opinion that besides teachers, health personnel and the police, a whole range of other stakeholders such as counsellors, government officials and policy makers need to be sensitized to make them empathic towards transgender persons and to overcome preconceived notions and biases that influences the ways in which they respond to transgender people.

### Recognising diversity among transgender people

Often all trans women are equated with *Hijras*, and it is assumed that all trans women are members of gharanas, which is not true. The hierarchical nature of the *Hijra gharana* is a cause of stress and may inhibit self-determination and autonomy of trans women living in *gharanas*. Similarly, trans women living in smaller towns and villages are forced to conceal their identity as these are conservative societies and therefore not supportive of gender nonconforming individuals ^41, 42^. This underlines the fact that even among trans gender communities there are some who are even more marginalized than others.

### Experience of structural stigma

Changes in policies that aim to reduce stigma and provide equal opportunities for trans gender people can positively influence their health ^2^. Studies have shown that government policies that did not extend protection to lesbian, gay, and bisexual individuals was associated with a higher magnitude of psychiatric disorders, particularly generalized anxiety disorder and post-traumatic stress disorder ^43^. Conversely protective policies were found to modify the effect of psychiatric disorders and improve the mental health of stigmatized groups ^43^. A positive move by the Supreme Court of India was its judgement on Article 377 that decriminalised what was earlier termed as unnatural sex. Following this the National Legal Services Authority (NALSA) judgement and transgender protection law were progressive steps taken by the Indian government but the implementation of these is minimal. Many other programmes have been announced, but implementation on the ground is patchy, resulting in stress.

### Research and policy implications

The issues that emerged from this study highlight concerns that policy makers and other relevant stakeholders including the research community must address, to promote the wellbeing of the transgender community.

 Researchers, especially cis-gender researchers must be cognizant of the fact that the responses given by transgender respondents might be affected by the self-stigma that they experience. To reduce the potential bias that this self-stigma might introduce, cis-gender researchers must try to make the transgender respondent as comfortable as possible before asking questions, especially questions that are sensitive in nature. Given the stigma that transgender people are subjected to and the adverse impacts that this has on their mental wellbeing, it is important for policy makers to address this by implementing special programmes and setting up services that are free and provide treatment without prejudice. The Transgender Persons (Protection of Rights) Rules, 2020 formulated and published in the Gazette of India on September 29, 2020 are a step in this direction. The rules recognise the identity of transgender people and prohibits discrimination in several areas such as education, employment, healthcare, property, holding public or private office, and access to and use of public services and benefits. In 2022, transgender people were included in a government health insurance scheme (Ayushman Bharat TG Plus Scheme) which purports to cover all aspects of transition-related healthcare for transgender people ^44^.

However, this alone is not enough. A study has shown that despite investing resources to improve the health of transgender people, they continue to experience discrimination and violence from cis-gender people ^45^. It is therefore pertinent for governments to invest in programmes that seek to change the attitude of cis-gender people through running anti-stigma campaigns. The media can play a crucial role in influencing people’s perception of transgender people. In the past media portrayals of transpersons reinforced and perpetuated stereotypes; however, in recent times a shift in media’s perception is discernible ^46^. This shift is welcome and may go a long way in reducing transphobia.

It is important to map key stakeholders in relation to transgender communities and ensure that they are included in community-based research with the transgender community. It is also important for researchers to be aware of the fact that like cis-gender people, transgender persons are not homogeneous; there are difference in their experiences as a result of their area of residence (rural vs urban), their class and their preferred gender. These are factors that researchers and policy makers should focus on while planning and designing research and policy interventions Currently, there is a lack of research on topics that the transgender community identifies as being important. It should be bridged urgently so that the policies that are formulated are more relevant to the needs of the transgender community. Given the extent of structural stigma that the community faces, it is critical to identify the sites where this plays out as well as how it affects them. This would involve not only conducting research to understand structural stigma better, but it would also involve designing programmes to address the stigma such as running anti-stigma campaigns. It is also important for such programmes to be reviewed and evaluated periodically.

## Limitations

This study was limited to trans women living in Kolkata and represents their experiences and perceptions. These might differ from the experiences and perceptions of trans women living elsewhere in India; and thus this study is limited in its generalisability. This study also does not deal with trans men or other gender and sexual minorities whose lived experiences might be rather different from that of the trans women living in Kolkata.

## Conclusion

To conclude, trans women face a continuous struggle because of their gender non-conformity and experience internal, interpersonal, and structural stigma. Researchers who are interested in conducting research among them, should be cognisant of the challenges they face and plan and design research that consciously seeks inputs from the community to ensure that the interventions are meaningful, have acceptability, and are executed in partnership with the community. The findings of such research will result in the identification of evidence-based strategies that will inform policies designed to address the challenges they face and promote the well-being of transgender people. 

## Ethics and consent

All procedures performed in studies involving human participants were in accordance with the ethical standards of The George Institute for Global Health, New Delhi, India (Ref. no. 15/2022). The study is in line with the 1964 Helsinki Declaration and its later amendments.

This study was approved by the Independent Ethics Committee of the George Institute for Global Health, New Delhi, India (Ref. no. 15/2022) on the 28 ^th^ July 2022.

The Participant Information Sheet and Consent Form (PISCF) had details about the purpose of conducting the research, participants rights as well as how the findings would be disseminated. Written informed consent has been obtained from all study participants. The PISCF was used to take written consent from all participants of FGDs and IDIs. Verbal consent was taken from workshop participants only

## Data Availability

Since transpersons are highly marginalized and vulnerable, and some of them have not come out, the data cannot be shared publicly even after deidentification, in the interest of their safety and wellbeing. Relevant anonymized data, metadata and the study protocol are available with the corresponding author and can be shared on receiving a request from anyone for research purposes, as part of a data-sharing agreement, once this paper is published. The participant information sheet and consent form as well as the focused group discussion and in-depth interview guides can be accessed from Zenodo: Study Instruments. https://doi.org/10.5281/zenodo.8153284 ^47^ Data are available under the terms of Creative Commons Attribution 4.0 International.
