# Supplementary material for: “Transgenders are not dinosaurs!” Stigma faced by transgender women in their daily lives in India: implications for research and policy
Source: Wellcome Open Res. 2024 Nov 12;9:496. Originally published 2024 Sep 3. [Version 2] doi: 10.12688/wellcomeopenres.22658.2 (PMC11541070; doi:10.12688/wellcomeopenres.22658.2)
Supplement: Peer review history

# Reviewer report: Reviewer response for version 2

**DOI:** 10.21956/wellcomeopenres.25804.r113653

**Published:** 2025-01-13

**Authors:** Manuela Barreto

**Affiliations:** 1 University of Exeter, Exeter, England, UK

This is a potentially interesting paper focusing on the lived experiences of trans women in Kolkata, India. While the analyses are worth publishing, I think the paper is not ready for indexing, especially because it is not adequately introduced. There are also some improvements to be made in the method and in the discussion sections. Below, I list my concerns in order of appearance in the text:

 -The introduction does not explain what research gap the present study addresses. In addition to including this, I would also recommend that the authors include a sentence to this effect in the background section of the abstract.

 -The aims stated in the ‘aims’ section seem to be those of the larger study from where the data was taken. Besides not relating to what is said in the introduction, these aims are not the aims of this specific paper. The authors need to refer broadly to the aims of the larger study and specifically to the aims of this study. There is a statement in the method section that appears to touch on this (“This article focuses on the lived experience of trans women and its implications on planning research among transgender women.”) but this needs to be better introduced. First, there is already some research on the lived experiences of trans women, so exactly what is the gap this work seeks to address? (maybe part of this is the India context, or the specific kinds of lived experiences you are interested in; these are fine aims, but do explain why it is worth while researching the experiences of trans women in this context over and above what is already known—e.g., what is it about the context of India, or Kolkata, more aptly, that might be different from the contexts where prior research has been conducted?). Second, the introductory paragraphs do not say anything about research with transgender women, so this needs to be mentioned—what is the problem you address? what do we already know and do not know about this?

 -The focus on transgender women is justified in the methods as: “This study was conducted with trans women only, as the collaborating partner, Kolkata Rista (KR), works almost exclusively with trans women.” This is very odd, given that the introduction only speaks about transgender women anyway. Sure, this might have been the reason, but since the introduction is only speaking about transgender women, this sentence seems unnecessary.

 -the method section should ideally include a statement of author positionality and there should be some reflection on how this might affect the study, data analysis and conclusions drawn.

 -participant recruitment: it seems this was volunteer, but this could be clearer. If so, I would suggest the text explicitly states that participants volunteered to participate.

 -I was confused about the long description of the hijra gharanas. The need for this became clear only after I read the results. I think a minor tweak to the text would make this clearer. For example, you could say that participants were trans women, some of whom lived in hijra gharanas—and then follow this with a description of what this entails.

 -could you say slightly more about how you went from codes to themes?

 -the last two themes in the results are very short and could be included within the structural stigma theme. Structural stigma is also about exclusion from decision making bodies, voicelessness, and lack of dedicated or safe spaces.

 -the discussion session repeats the results too much. It should include a brief summary of the findings and then truly discuss them by reference to what is already known, what this adds to existing knowledge, and what implications it might have.

 -the limitations section is good, but I would recommend adding a couple of sentences reflecting on how and why living in Kolkata might differ from living elsewhere in India, for trans women.

Is the work clearly and accurately presented and does it cite the current literature?

Partly

If applicable, is the statistical analysis and its interpretation appropriate?

Not applicable

Are all the source data underlying the results available to ensure full reproducibility?

Partly

Is the study design appropriate and is the work technically sound?

Partly

Are the conclusions drawn adequately supported by the results?

Partly

Are sufficient details of methods and analysis provided to allow replication by others?

Partly

Reviewer Expertise:

I am a social psychologist, expert on social stigma, and have done very similar research to the one reported here with a range of stigmatized groups, including transgender people.

I confirm that I have read this submission and believe that I have an appropriate level of expertise to state that I do not consider it to be of an acceptable scientific standard, for reasons outlined above.

# Reviewer report: Reviewer response for version 2

**DOI:** 10.21956/wellcomeopenres.25804.r114728

**Published:** 2024-12-19

**Authors:** Ximena Salazar

**Affiliations:** 1 Universidad Peruana Cayetano Heredia, Miraflores, Peru

The article is well-crafted. The results reached by the researchers are not new and can be corroborated with similar findings in many parts of the world, as the situation of trans women: stigma, discrimination, exclusion, and lack of opportunities today are similar. What I find lacking in this article is an exploration of how this situation has developed in India, considering that Hijras were respected figures in previous times. It is needed a critique of the colonization period because it is from there that everything changes, with Western morals, leading to a commodification of Hijra tasks and power dynamics among them and the contempt for the role of trans women. In summary, I would like to see reflected in the article how and why the trans community has arrived at its current situation in Kolkata.

Is the work clearly and accurately presented and does it cite the current literature?

Yes

If applicable, is the statistical analysis and its interpretation appropriate?

Not applicable

Are all the source data underlying the results available to ensure full reproducibility?

Yes

Is the study design appropriate and is the work technically sound?

Yes

Are the conclusions drawn adequately supported by the results?

Partly

Are sufficient details of methods and analysis provided to allow replication by others?

Yes

Reviewer Expertise:

My research area focuses on gender and sexual diversity topics, especially the social and health situation of the transgender male and female population, as well as community strengthening issues. I am an expert in qualitative research.

I confirm that I have read this submission and believe that I have an appropriate level of expertise to confirm that it is of an acceptable scientific standard, however I have significant reservations, as outlined above.

# Response

**Published:** 2024-12-22

**Authors:** Kanaka Sandhya Yatirajula

**Affiliations:** Mental Health, The George Institute for Global Health India, New Delhi, Delhi, India

*We thank the reviewer for their comment and agree that it is an important area worthy of exploration. However, we are unable to provide greater details related to why and how the Hijras community’s status declined over the colonial period, as it is beyond the scope of the research on which this paper is based. We hope that we or some other researchers will be able to do justice to this important area in subsequent studies*.

# Reviewer report: Reviewer response for version 1

**DOI:** 10.21956/wellcomeopenres.24959.r98817

**Published:** 2024-11-06

**Authors:** Reya Farber

**Affiliations:** 1 William & Mary, Williamsburg, USA

Thank you for the opportunity to review this important paper. This is a clear and well-written article that expands empirical and theoretical knowledge of transgender women's stigma in India. It charts necessary next steps for more social-ecological research on trans people, as well as implications for policy.

 My only suggestion is that the authors consider moving the following segment, which seems like it would be helpful context earlier in the paper (perhaps in the participant selection portion of the Methods): “Some of the study participants were or had been members of *Hijra gharanas.* The *hijra* community in India are systematically organized as hierarchical communities within themselves, which are called *gharanas*. The word *hijra* is traditionally used to refer to trans women or intersex persons. It should be noted however that not all trans women identify themselves as or belong to the *hijra* community. The word *gharana means* household/community. Traditionally the *hijra* community visit a household during weddings or birth of a child to perform dances and give blessings and get money from the family in return. Each *gharana* is headed by a *Guru* (leader), who assumes the status of ‘mother’; the status is achieved when a senior trans woman receives a newly joined trans woman as her *chela* (follower/disciples). The *chela* has to serve the *Guru* and offer a certain portion of her daily earnings to her *Guru*. The *Guru* is the caretaker of her c *helas and* members of a gharana lived together under the patronage of their *Guru.*”

Is the work clearly and accurately presented and does it cite the current literature?

Yes

If applicable, is the statistical analysis and its interpretation appropriate?

Not applicable

Are all the source data underlying the results available to ensure full reproducibility?

No

Is the study design appropriate and is the work technically sound?

Yes

Are the conclusions drawn adequately supported by the results?

Yes

Are sufficient details of methods and analysis provided to allow replication by others?

Yes

Reviewer Expertise:

Medical sociology, gender studies, globalization

I confirm that I have read this submission and believe that I have an appropriate level of expertise to confirm that it is of an acceptable scientific standard.

# Response

**Published:** 2024-11-08

**Authors:** Kanaka Sandhya Yatirajula

**Affiliations:** Mental Health, The George Institute for Global Health India, New Delhi, Delhi, India

We thank the reviewers for reviewing our article. As recommended by the second reviewer, we have shifted the description of the Hijra Gharanas from the Findings to the Methods section under the Participant Selection sub-heading.

# Reviewer report: Reviewer response for version 1

**DOI:** 10.21956/wellcomeopenres.24959.r99656

**Published:** 2024-10-02

**Authors:** Danish Suleman

**Affiliations:** 1 Department of English Language and Literature, Near East University, Lefkosa, Nicosia, Cyprus

The source data is not included here. However, the author has mentioned that it can be provided by the corresponding author upon request.

 Overall, the article's method and analysis fulfilled the research objectives and explained the process clearly.

 The stigma highlighted by the authors is shown positively in the analysis section.

Is the work clearly and accurately presented and does it cite the current literature?

Yes

If applicable, is the statistical analysis and its interpretation appropriate?

Not applicable

Are all the source data underlying the results available to ensure full reproducibility?

No

Is the study design appropriate and is the work technically sound?

Yes

Are the conclusions drawn adequately supported by the results?

Yes

Are sufficient details of methods and analysis provided to allow replication by others?

Yes

Reviewer Expertise:

gender studies; social structures; social stigma; qualitative methods; thematic analysis

I confirm that I have read this submission and believe that I have an appropriate level of expertise to confirm that it is of an acceptable scientific standard.
